# Supplementary material for: Transcriptomic sex differences in early human fetal brain development
Source: Commun Biol. 2025 Apr 25;8:664. doi: 10.1038/s42003-025-08070-3 (PMC12032161; doi:10.1038/s42003-025-08070-3)
Supplement: Supplementary file 1 — Supplementary Information [file 42003_2025_8070_MOESM1_ESM.pdf]

Supplementary Information

Transcriptomic sex differences in early human fetal brain development

Federica Buonocore[1*], Jenifer P Suntharalingham[1], Olumide K Ogunbiyi[2,3], Aragorn Jones[4], Nadjeda Moreno[3], Paola Niola[5], Tony Brooks[5], Nita Solanky[3], Mehul T. Dattani[1], Ignacio del Valle[1†], John C. Achermann[1*†]

[1]Genetics and Genomic Medicine Research and Teaching Department, UCL Great Ormond Street Institute of Child Health, University College London, London, WC1N 1EH, United Kingdom

[2]Department of Histopathology, Great Ormond Street Hospital for Children National Health Service (NHS) Foundation Trust, London, WC1N 3JH, United Kingdom

[3]Developmental Biology and Cancer Research and Teaching Department, UCL Great Ormond Street Institute of Child Health, University College London, London, WC1N 1EH, United Kingdom

[4]Biosciences Institute, Faculty of Medical Sciences, Newcastle University, Newcastle upon Tyne, NE2 4HH, United Kingdom

[5]UCL Genomics, Zayed Centre for Research, UCL Great Ormond Street Institute of Child Health, University College London, London WC1N 1DZ, United Kingdom

Corresponding author: f.buonocore@ucl.ac.uk

List of Supplementary Table and Figures

Supplementary Table 1. Expression of Y chromosome genes in human adult tissues

Supplementary Figure 1. Principal component analysis (PCA) showing effect of karyotype in non-brain control tissues.

Supplementary Figure 2. Differential expression of *PCDH11Y* in a panel of adult tissues.

Supplementary Figure 3. Comparative genomics of *PCDH11X*/*PCDH11Y* in humans with 185 speciation nodes.

Supplementary Figure 4. Differential expression of *PCDH11X* in a panel of adult tissues.

Supplementary Figure 5. Genomic locus of the long non-coding RNA, *RP11-424G14.1*

Supplementary Figure 6. Expression of Y chromosome genes in human fetal brain cortex samples compared to control tissues

Supplementary Figure 7. Differential expression of the gene encoding the androgen receptor (*AR*) in a panel of adult tissues.

Supplementary Figure 8. Number of differentially expressed genes (DEGs) (46,XX versus 46,XY) in the Brain-Seq 1 and Brain-Seq 2 datasets with developmental stage.

Supplementary Figure 9. Analyses of genes showing changes in the period of biological androgen exposure in Brain-Seq 1 and Brain-Seq 2 datasets.

Supplementary Figure 10. Expression of the ten 46,XY specific common DEGs identified in the period of biological androgen exposure.

Supplementary Figure 11. Analyses of genes showing changes in the period of biological androgen exposure in Brain-Seq 1 and Brain-Seq 2 datasets.

Supplementary Figure 12. Venn diagrams showing the intersection of AR interactors with Brain-Seq differentially expressed genes (DEGs).

Supplementary Figure 13. Expression of genes involved in the classic steroid pathway in the human brain cortex during early fetal development.

Supplementary Figure 14. Expression of genes involved in the backdoor steroid pathway in the human brain cortex during early fetal development.

Supplementary Figure 15. Expression of genes encoding aromatase (*CYP19A1*) and the estrogen receptors (*ESR1*, *ESR2*) in the human brain cortex during early fetal development.

Supplementary Figure 16. Androgen receptor expression by immunohistochemistry in the developing diencephalon and cerebral cortex at 10 weeks post conception.

Supplementary Figure 17. Nuclei annotations of single-cell RNA-seq data in the human pituitary.

Supplementary Figure 18. Nuclei annotations of single-cell RNA-seq data in the human hypothalamus.

Supplementary Figure 19. MultiQC analysis for the Brain-Seq 1 dataset.

Supplementary Table 1

Brain-enriched Y chromosome genes	RNA tissue specificity (consensus dataset)
AC009977.1	na
AC010970.2	na
ACTG1P2	na
AGKP1	na
AMELY	not detected
ANKRD36P1	na
ANOS2P	na
ARSFP1	na
BCORP1	na
CD99	Low tissue specificity
DDX3Y	Tissue enhanced (bone marrow)
EIF1AY	Tissue enhanced (heart muscle, tongue)
FAM8A4P	na
GPR143P	na
GYG2P1	na
HSFY2	Tissue enriched (testis)
HSFY3P	na
KDM5D	Low tissue specificity
MED14P1	na
NAP1L1P2	na
NLGN4Y	Tissue enhanced (seminal vesicle)
P2RY8	Group enriched (bone marrow, lymphoid tissue)
PARPAP1	na
PCDH11Y	Tissue enhanced (brain)
PRKY	na
RFTN1P1	na
RNU2-57P	na
RP11-424G14.1	na
RP11-576C2.1	na
RPS4Y1	Low tissue specificity
RPS4Y2	Group enriched (prostate, testis)
SHOX	Low tissue specificity
SRY	Tissue enhanced (skin, testis)
TAB3P1	na
TBL1Y	Tissue enhanced (prostate, thyroid gland)
TMSB4Y	Tissue enhanced (intestine)
TPTE2P4	na
TSPY2	Tissue enriched (testis)
TTTY10	na
TTTY14	na
TTTY15	na
TXLNGY	na
USP9Y	Low tissue specificity
USP9YP14	na
USP9YP28	na
UTY	Low tissue specificity
XG	Tissue enhanced (adipose tissue, skin)
ZFY	Low tissue specificity
ZFY-AS1	na

Supplementary Table 1. Expression of Y chromosome genes in human adult tissues. Data were obtained from the Human Protein Atlas "Consensus" dataset for bulk RNA-sequencing (50 tissues, including 10 brain/neuronal regions). The brain specific expression of *PCDH11Y* is indicated in red. na, not available.

Supplementary Figure 1

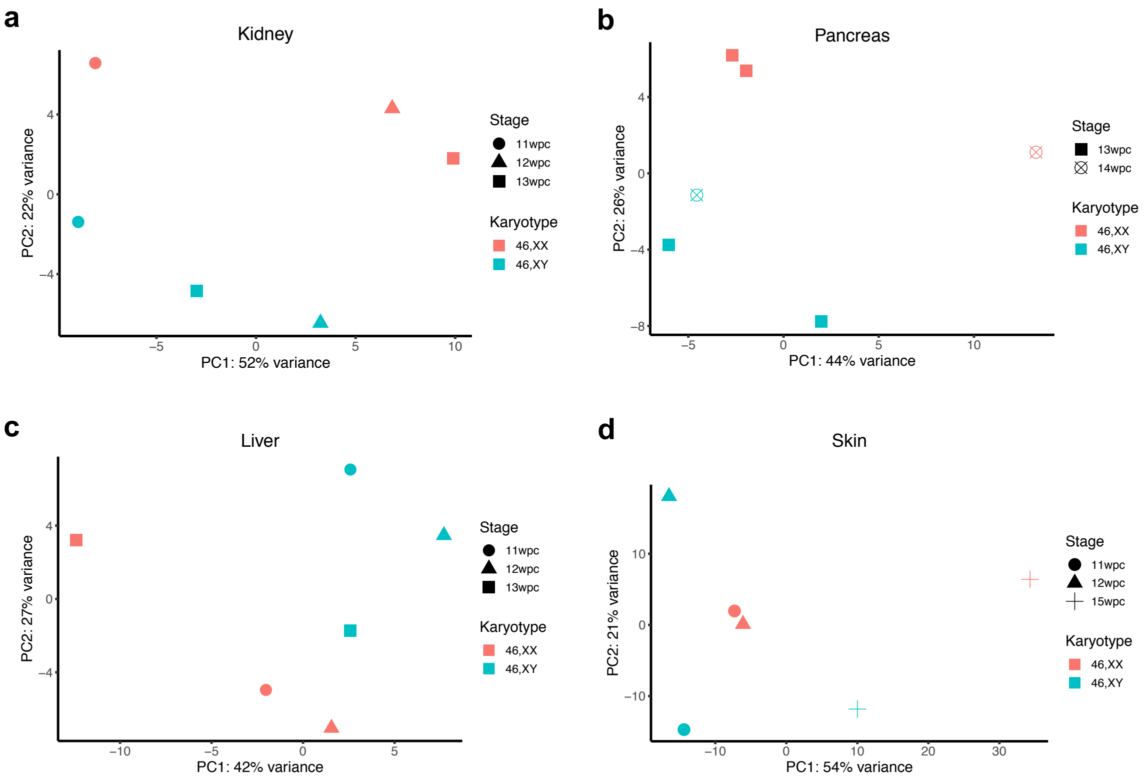

Supplementary Figure 1. Principal component analysis (PCA) showing effect of karyotype in non-brain control tissues. Data for bulk RNA-sequencing of kidney, pancreas, liver and skin are shown (n=6 each group). Three 46,XX and three 46,XY samples were included for each tissue, matched for developmental stage, as indicated. PC, principal component.

Supplementary Figure 2

a

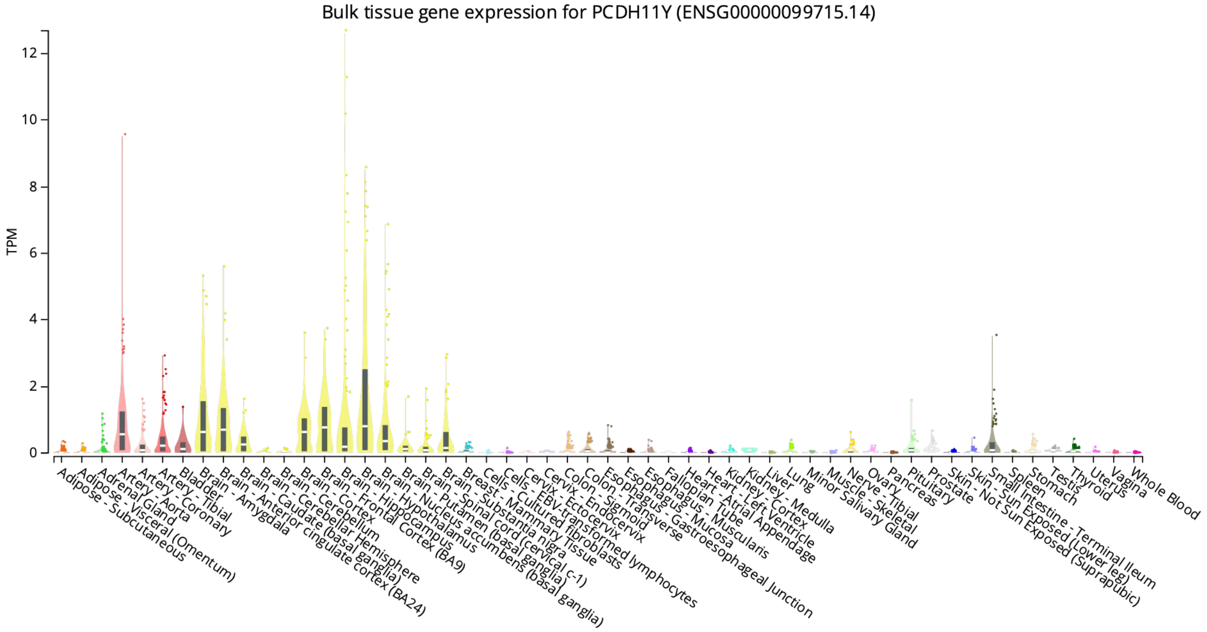

b

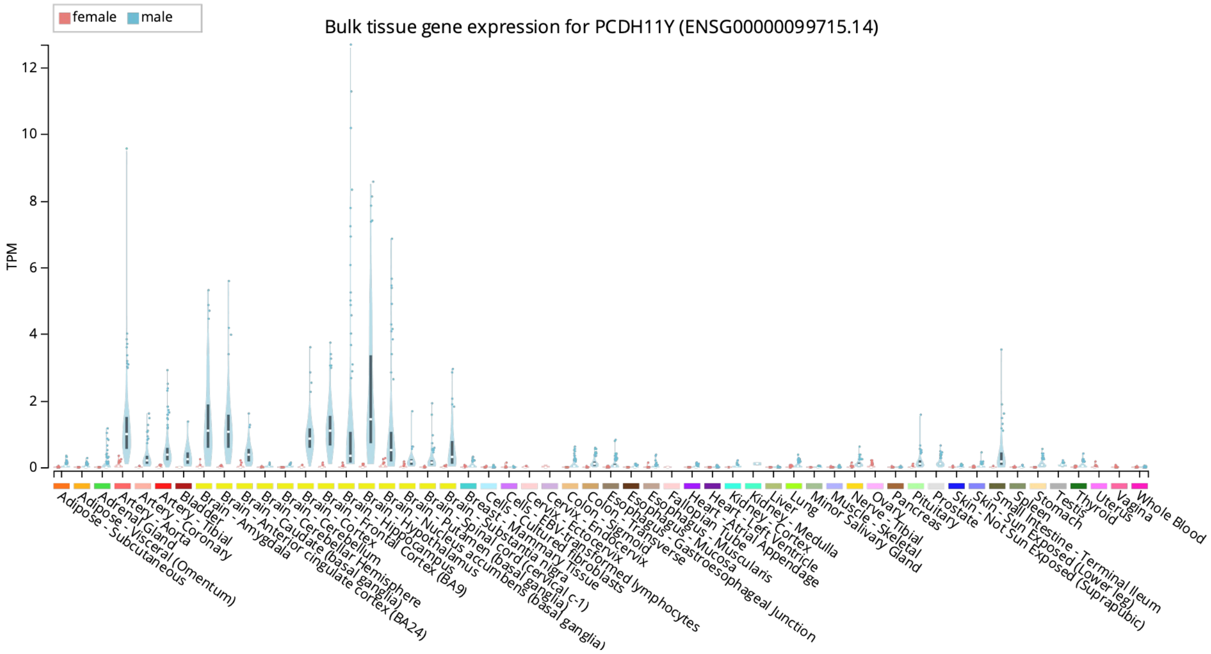

Supplementary Figure 2. Differential expression of *PCDH11Y* in a panel of adult tissues. a *PCDH11Y* expression in all samples. **b** *PCDH11Y* expression shown by sex. Data represent bulk RNA-sequencing from the Genotype-Tissue Expression (GTEx) Project. GTEx was supported by the Common Fund of the Office of the Director of the National Institutes of Health, and by NCI, NHGRI, NHLBI, NIDA, NIMH, and NINDS. The data were obtained from the GTEx Portal on 10/10/23 (GTEx Analysis Release V8; dbGaP Accession phs000424.v8.p2). TPM, transcripts per million.

Supplementary Figure 3

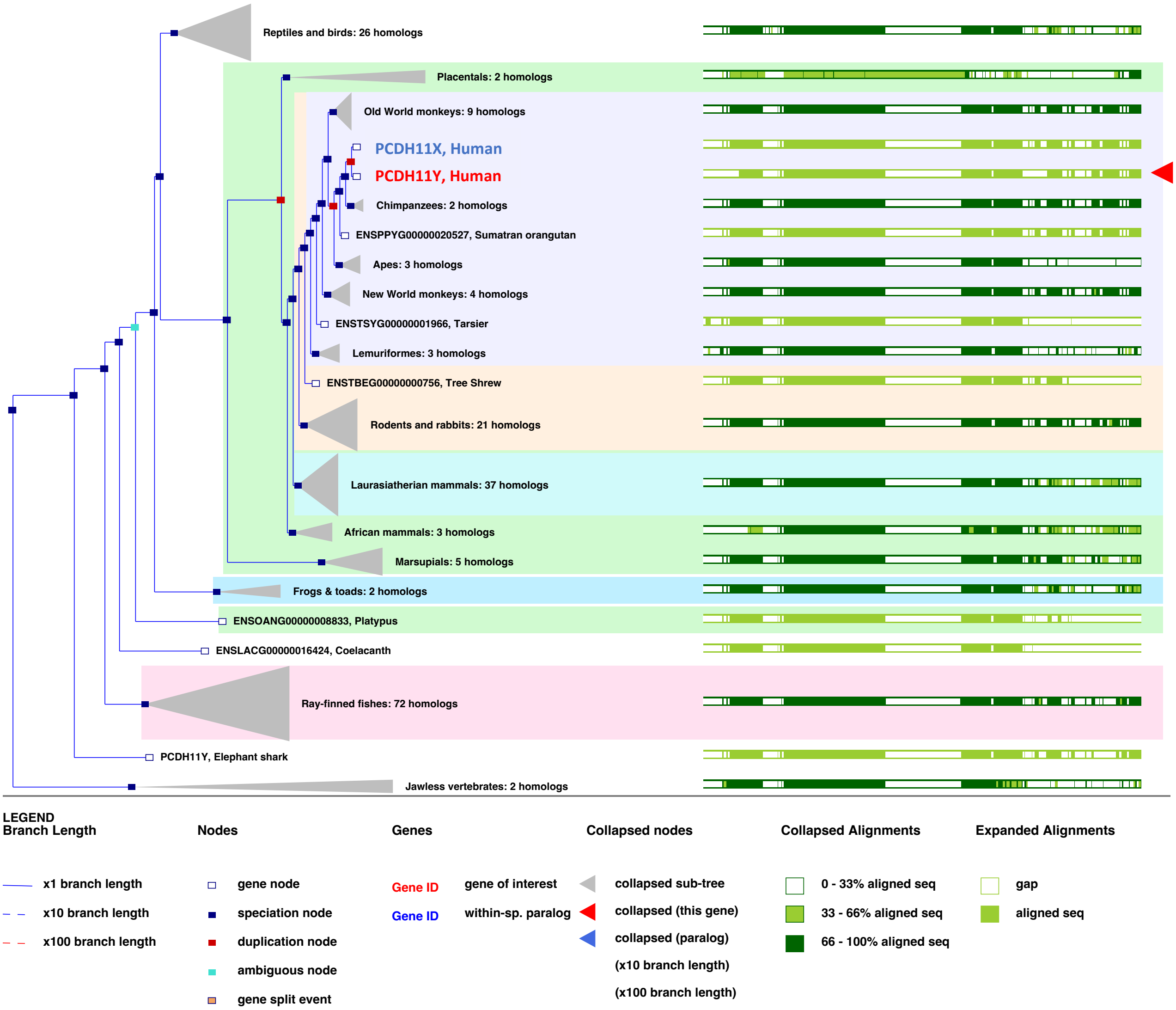

Supplementary Figure 3. Comparative genomics of *PCDH11X*/*PCDH11Y* in humans with 185 speciation nodes. The human *PCDH11X* transcript is shown in blue and has strong alignment with key domains of the paralogue in other species. The human *PCDH11Y* transcript is shown in red and shows localized regions of variance. Data derived and reproduced from GeneTree (ENSGT00940000158335) in Ensembl Genome Browser release 110 (https://www.ensembl.org/) (Martin FJ, et al. Nucleic Acids Res. 2023, 51(D1):D933-D941; doi:10.1093/nar/gkac958).

Supplementary Figure 4

a

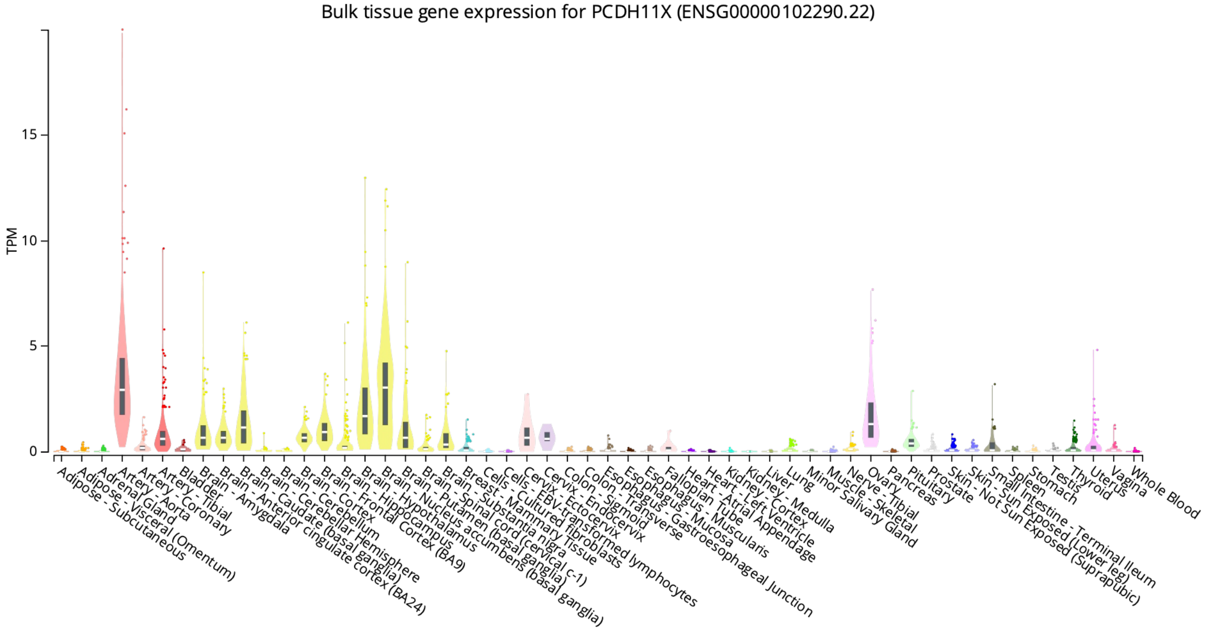

b

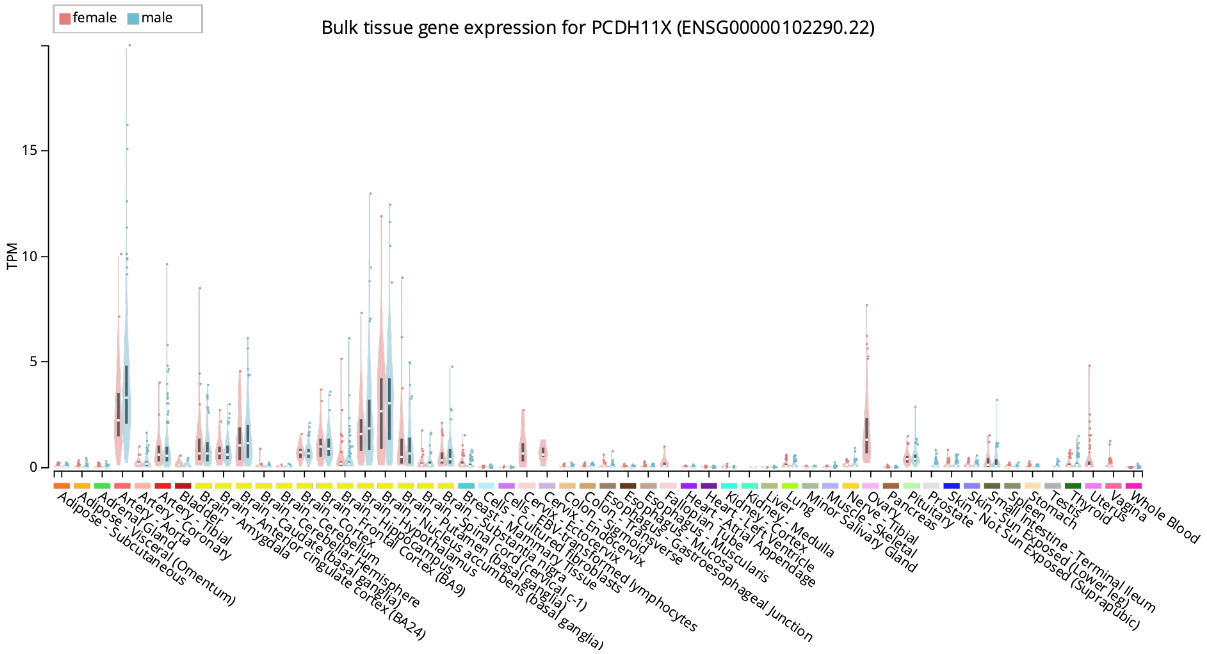

Supplementary Figure 4. Differential expression of *PCDH11X* in a panel of adult tissues. a *PCDH11X* expression in all samples. **b** *PCDH11X* shown by sex. Data represent bulk RNA-sequencing from the Genotype-Tissue Expression (GTEx) Project. GTEx was supported by the Common Fund of the Office of the Director of the National Institutes of Health, and by NCI, NHGRI, NHLBI, NIDA, NIMH, and NINDS. The data were obtained from the GTEx Portal on 10/10/23 (GTEx Analysis Release V8; dbGaP Accession phs000424.v8.p2). TPM, transcripts per million.

Supplementary Figure 5

a

RP11-424G14.1 (ENSG00000260197) (Chr Y:19,691,941-19,694,606)

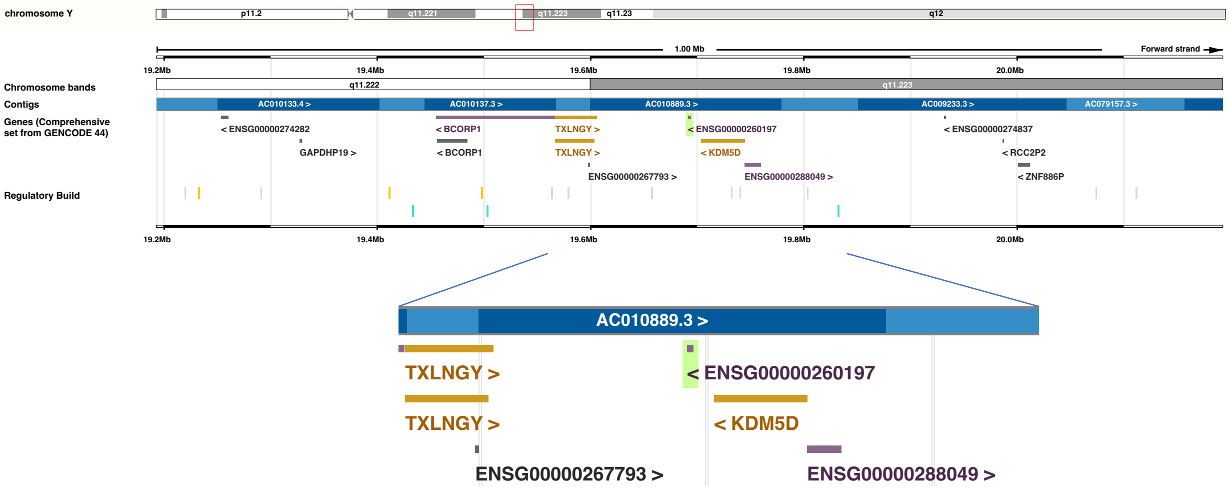

b

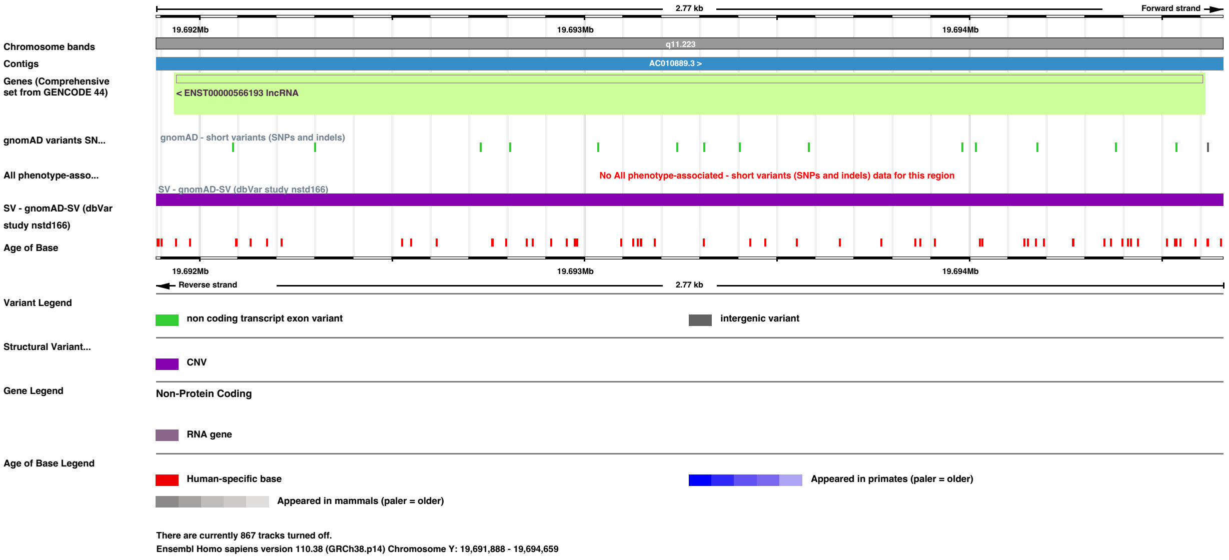

Supplementary Figure 5. Genomic locus of the long non-coding RNA, *RP11-424G14.1*. a Location of *RP11-424G14.1* (shown as Ensembl gene ENSG00000260197) on the long arm of the Y chromosome in minus orientation and centromeric to *KDM5D*. b Locus characteristics of the transcript (ENST00000566193.1) associated with *RP11-424G14.1.* Data derived and reproduced from Ensembl release 110 (https://www.ensembl.org/) (Martin FJ, et al. Nucleic Acids Res. 2023, 51(D1):D933-D941; doi:10.1093/nar/gkac958).

Supplementary Figure 6

a

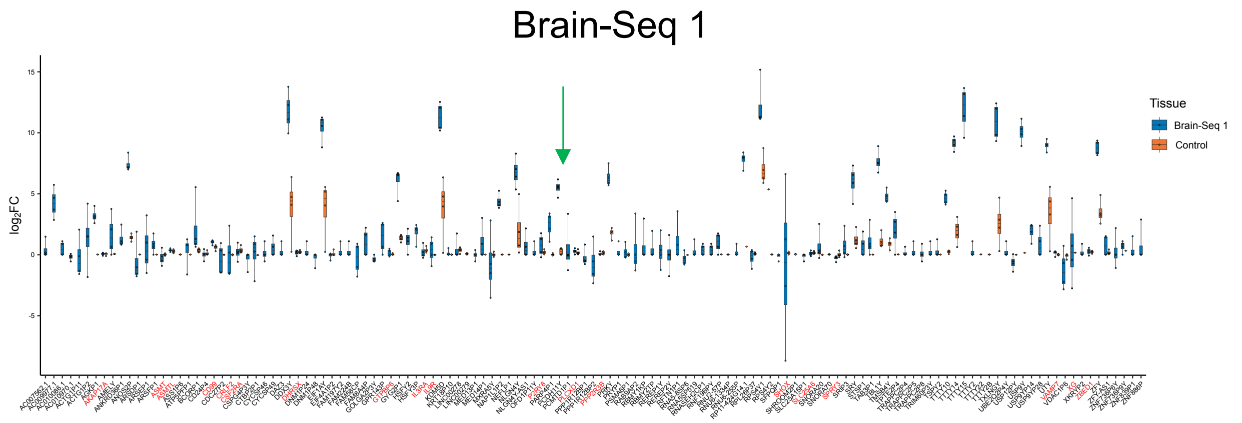

b

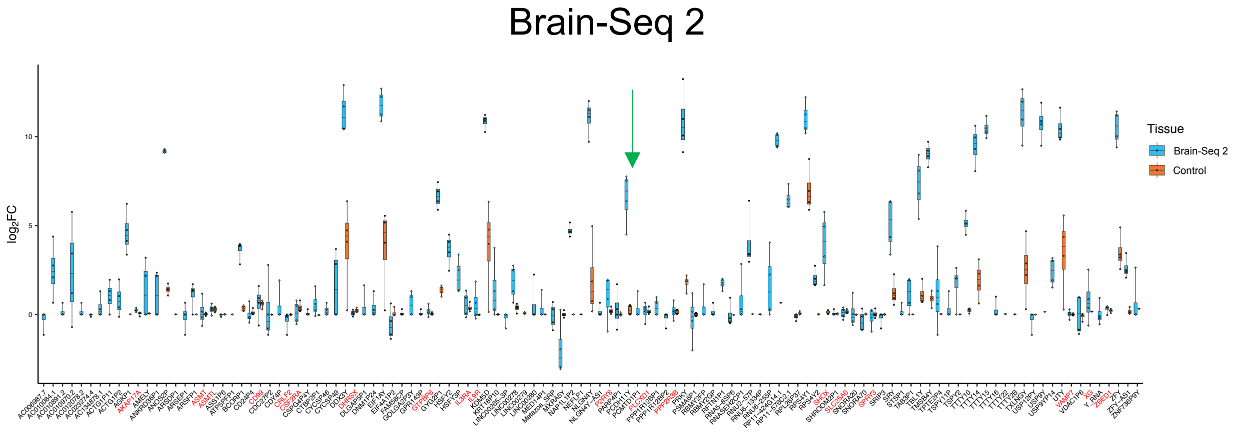

Supplementary Figure 6. Expression of Y chromosome genes in human fetal brain cortex samples compared to control tissues. a Brain-Seq 1 dataset. **b** Brain-Seq 2 dataset**.** Data are shown for bulk RNA-sequencing as log2 fold change (FC) of Y chromosome genes following comparison of 46,XY vs 46,XX brain samples and 46,XY vs 46,XX control samples. Each brain dataset consisted of n=32 cortex samples between CS22/23 and 15-17 weeks post conception (wpc). Control dataset consisted of n=32 samples including skin, pancreas, liver and kidney tissues between 11 and 15 wpc (each tissue having n=8). Boxplots represent the distribution of log2FC values of each gene, with the box extending from the first to the third quartile and the median shown with a line. Genes labelled in red are in pseudoautosomal regions (PAR). FC, fold change. Green arrows indicate the position of *PCDH11Y.*

Supplementary Figure 7

a

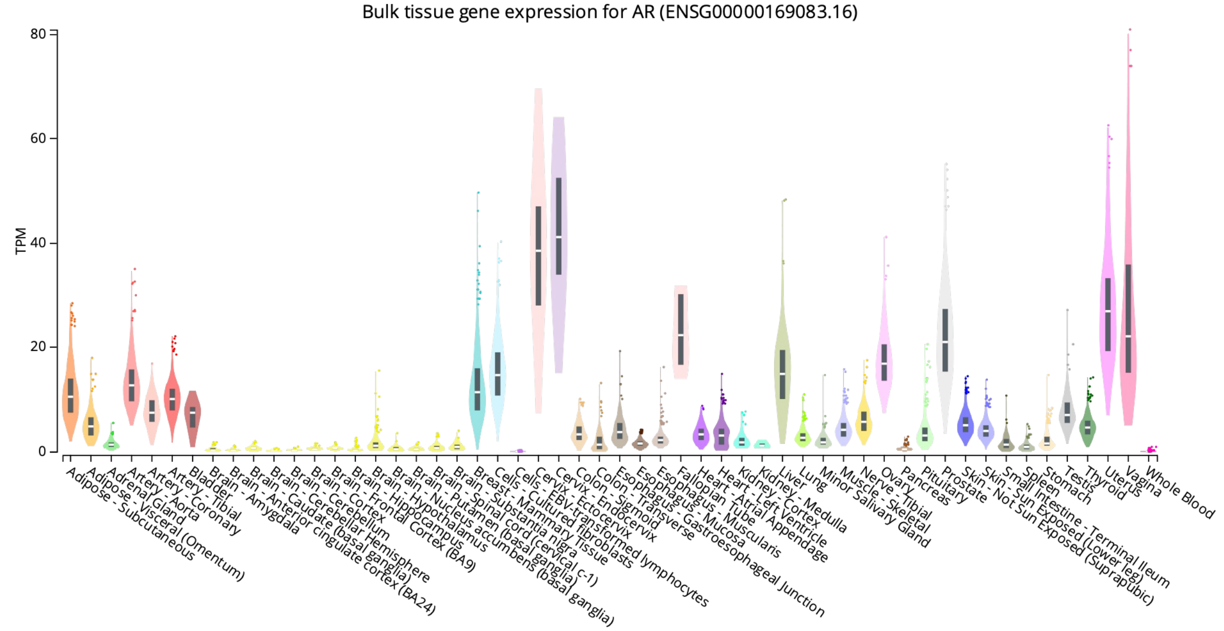

b

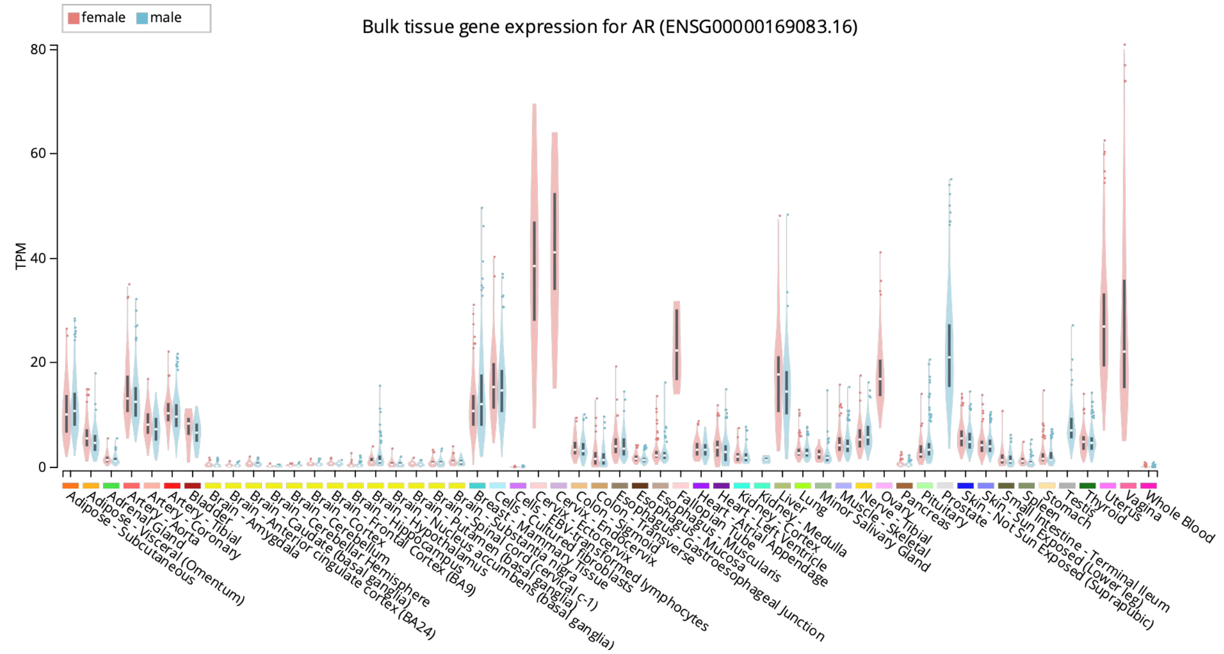

Supplementary Figure 7. Differential expression of the gene encoding the androgen receptor (*AR*) in a panel of adult tissues. a *AR* expression in all samples. **b** *AR* expression shown by sex. Data represent bulk RNA-sequencing from the Genotype-Tissue Expression (GTEx) Project. GTEx was supported by the Common Fund of the Office of the Director of the National Institutes of Health, and by NCI, NHGRI, NHLBI, NIDA, NIMH, and NINDS. The data were obtained from the GTEx Portal on 10/10/23 (GTEx Analysis Release V8; dbGaP Accession phs000424.v8.p2). TPM, transcripts per million.

Supplementary Figure 8

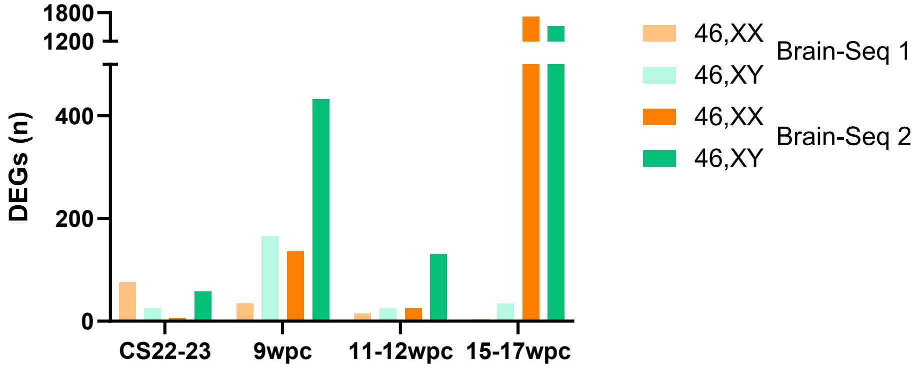

Supplementary Figure 8. Number of differentially expressed genes (DEGs) (46,XX versus 46,XY) in the Brain-Seq 1 and Brain-Seq 2 datasets with developmental stage. All DEGs in both 46,XX and 46,XY samples with a p-adj <0.05 were used. DEGs, differentially expressed genes; wpc, weeks post conception

Supplementary Figure 9

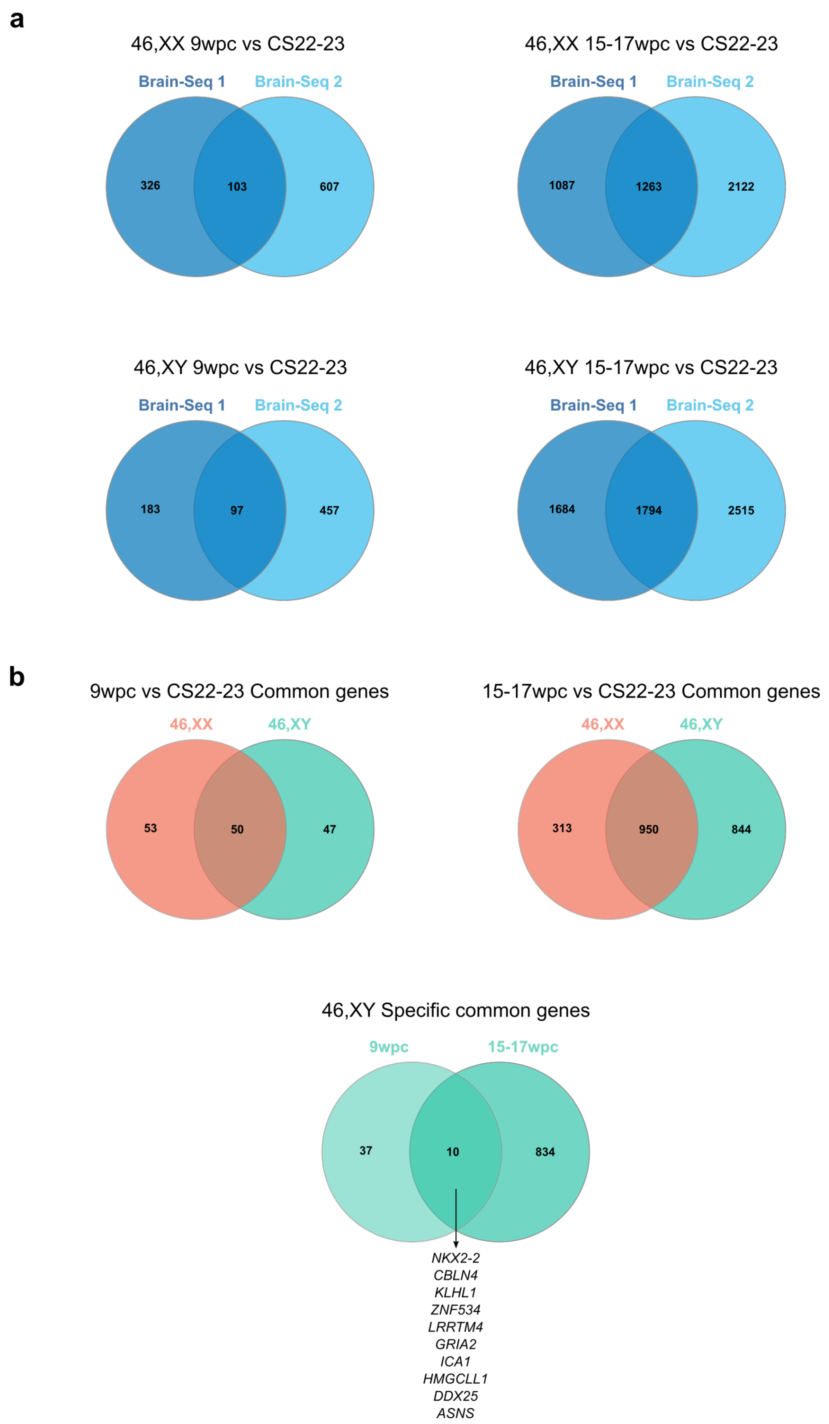

Supplementary Figure 9. Analyses of genes showing changes in the period of biological androgen exposure in Brain-Seq 1 and Brain-Seq 2 datasets. a Differentially expressed genes (DEGs) in both 46,XX and 46,XY comparing 9wpc and CS22-23, and 15-17wpc and CS22-23. **b** DEGs common to both Brain-Seq datasets in 9wpc versus CS22-23 (46,XX 9wpc versus CS22-23, n=103; 46,XY 9wpc versus CS22-23, n=97) and 15-17wpc versus CS22-23 (46,XX 15-17wpc versus CS22-23, n=1263; 46,XY 15-17wpc versus CS22-23, n=1794). Ten 46,XY specific common DEGs were identified. vs, versus.

Supplementary Figure 10

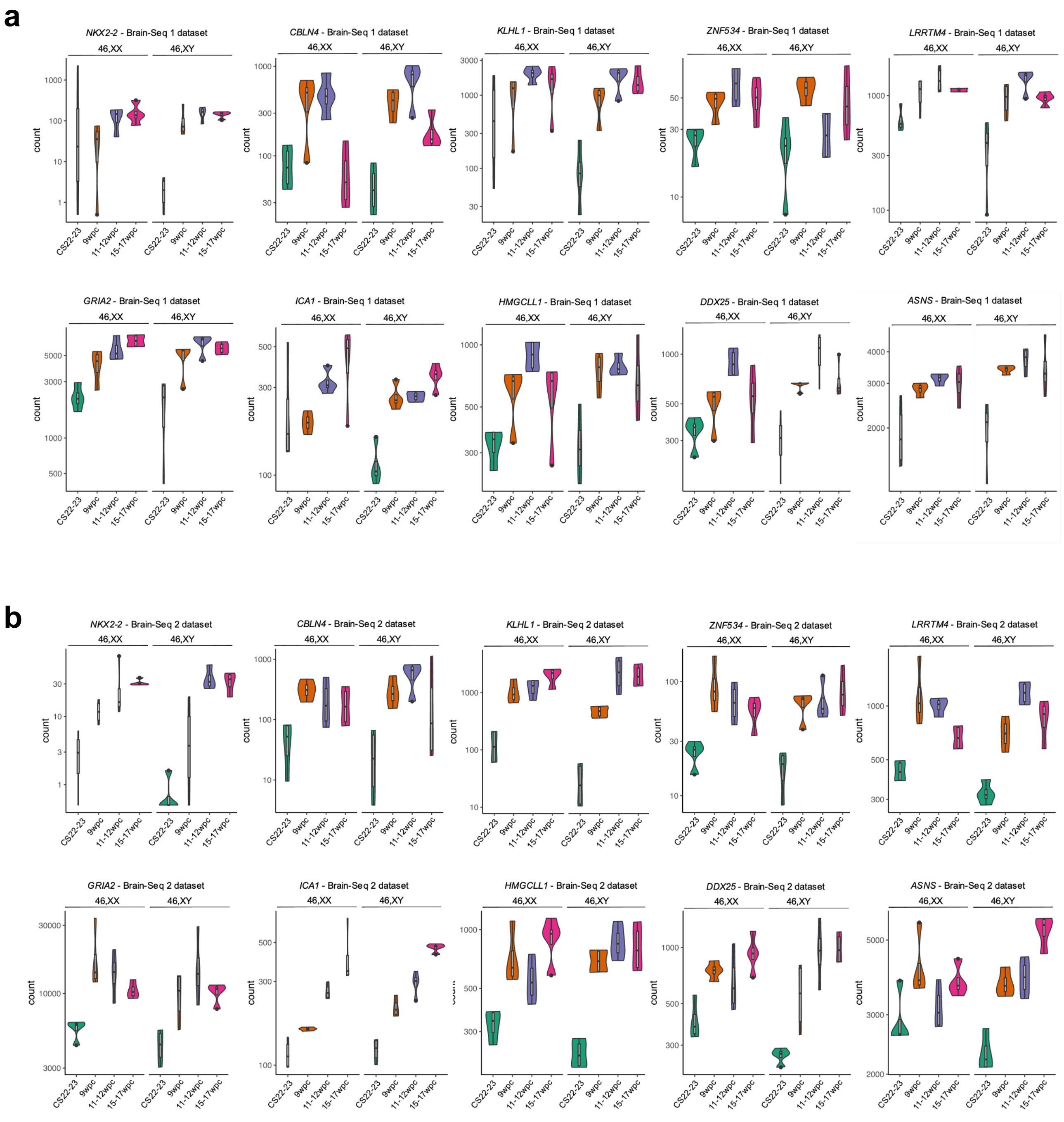

Supplementary Figure 10. Expression of the ten 46,XY specific common DEGs identified in the period of biological androgen exposure. a Brain-Seq 1 dataset. **b** Brain-Seq 2 dataset. Data are shown as violin plots of normalized counts on a log scale, with n=4 in each group. CS, Carnegie stage; wpc, weeks post-conception.

Supplementary Figure 11

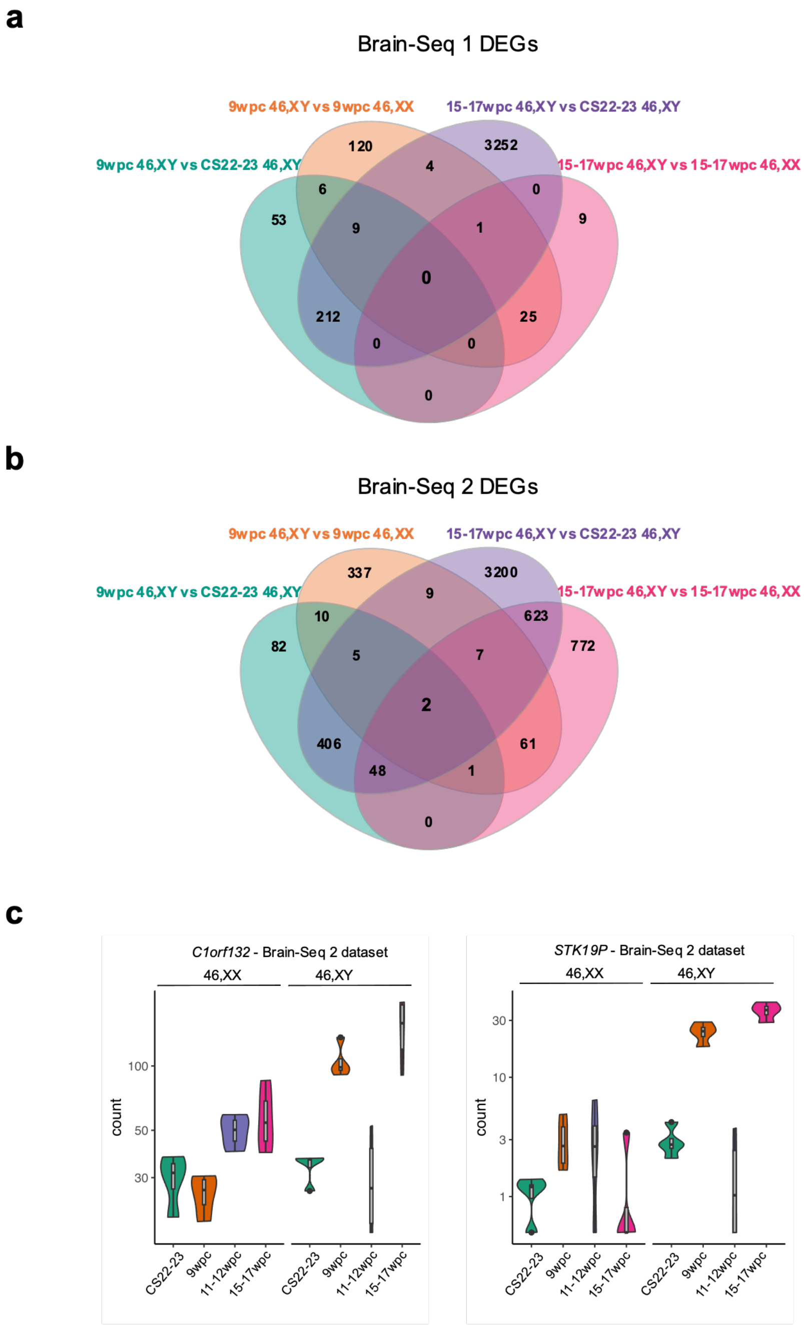

Supplementary Figure 11. Analyses of genes showing changes in the period of biological androgen exposure in Brain-Seq 1 and Brain-Seq 2 datasets. a Brain-Seq 1 dataset. **b** Brain-Seq 2 dataset. **c** The two common genes in Brain-Seq 2 dataset. Data are shown as violin plots of normalized counts on a log scale, with n=4 in each group. CS, Carnegie stage; wpc, weeks post-conception.

Supplementary Figure 12

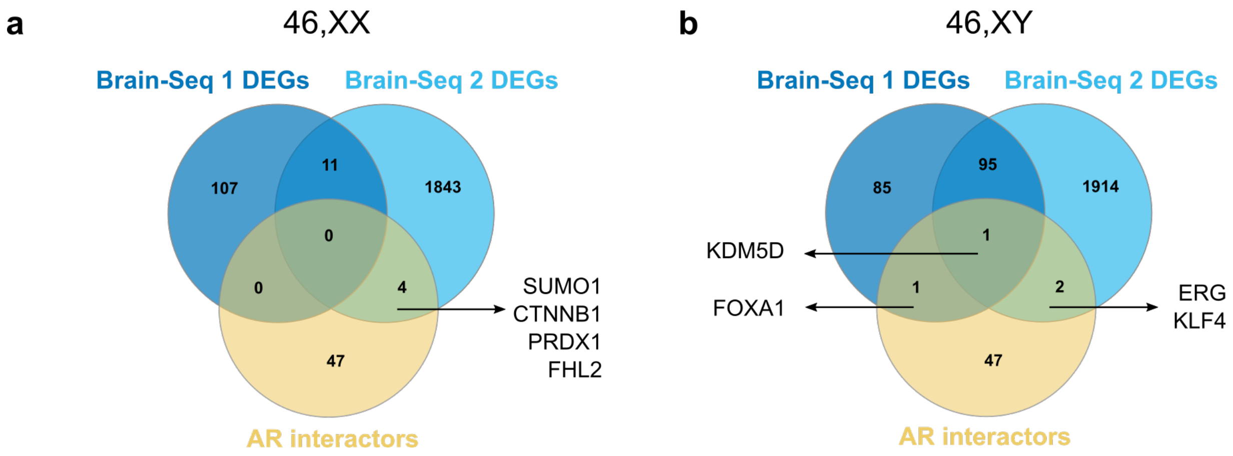

Supplementary Figure 12. Venn diagrams showing the intersection of AR interactors with Brain-Seq differentially expressed genes (DEGs). a Overlap of DEGs in both 46,XX datasets with AR interactors. **b** Overlap of DEGs in both 46,XX datasets with AR interactors. AR interactors are reported proteins that interact with the androgen receptor as listed in the Reactome Knowledgebase (https://reactome.org) (Gillespie et al., 2022).

Supplementary Figure 13

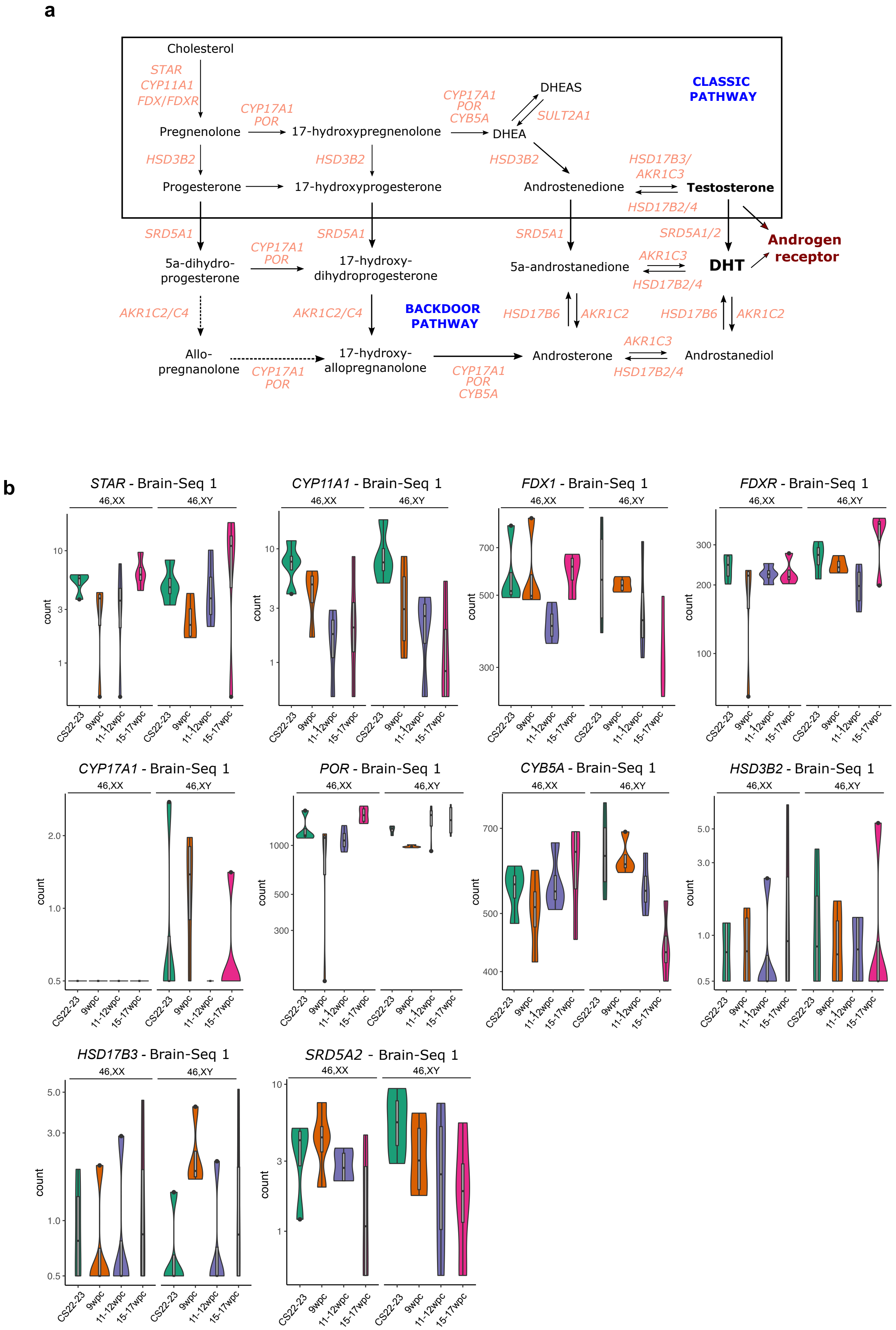

Supplementary Figure 13. Expression of genes involved in the classic steroid pathway in the human brain cortex during early fetal development. **a** Classic steroid biosynthesis pathway. Adapted from del Valle I et al., JCI Insight 2023;8(14):e168177; doi.org/10.1172/jci.insight.168177. **b** Data are shown as violin plots of normalized counts on a log scale, with n=4 in each group. CS, Carnegie stage; wpc, weeks post-conception.

Supplementary Figure 14

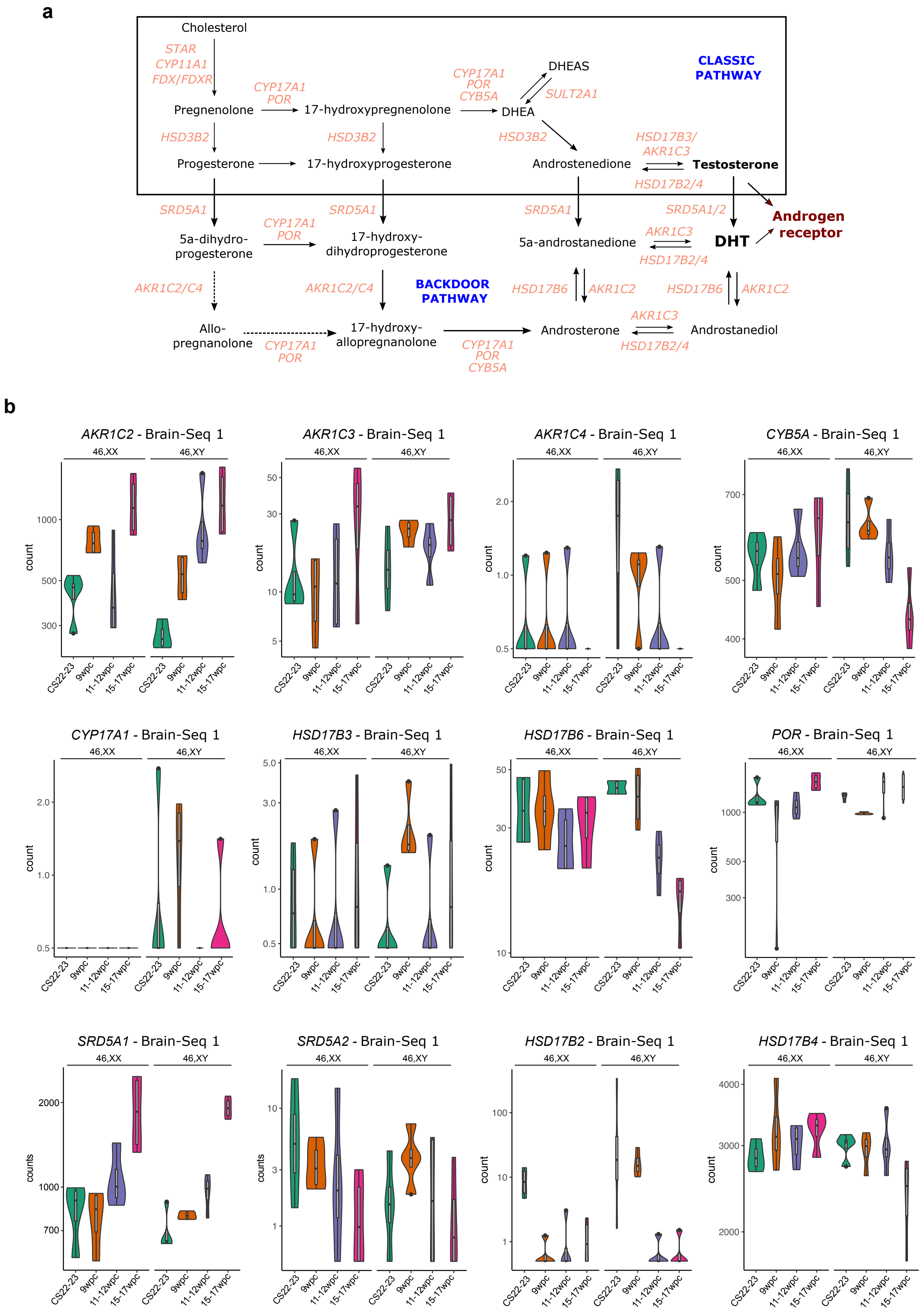

Supplementary Figure 14. Expression of genes involved in the backdoor steroid pathway in the human brain cortex during early fetal development. a Classic and proposed backdoor steroid biosynthesis pathway. Adapted from del Valle I et al., JCI Insight 2023;8(14):e168177; doi.org/10.1172/jci.insight.168177. **b** Data are shown as violin plots of normalized counts on a log scale, with n=4 in each group. CS, Carnegie stage; wpc, weeks post-conception.

Supplementary Figure 15

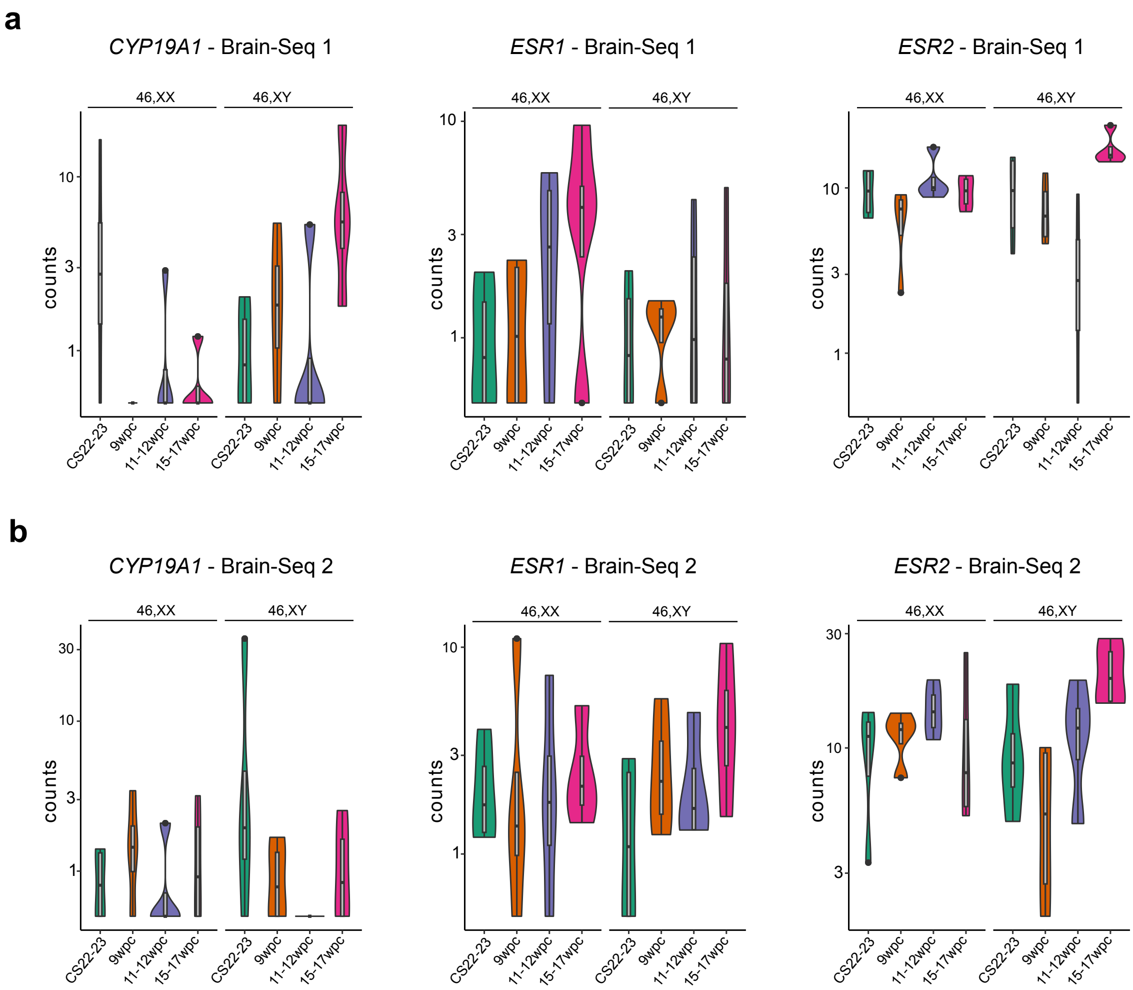

Supplementary Figure 15. Expression of genes encoding aromatase (*CYP19A1*) and the estrogen receptors (*ESR1*, *ESR2*) in the human brain cortex during early fetal development. a Brain-Seq 1 dataset. **b** Brain-Seq 2 dataset. Data are shown as violin plots of normalized counts on a log scale, with n=4 in each group. CS, Carnegie stage; wpc, weeks post-conception.

Supplementary Figure 16

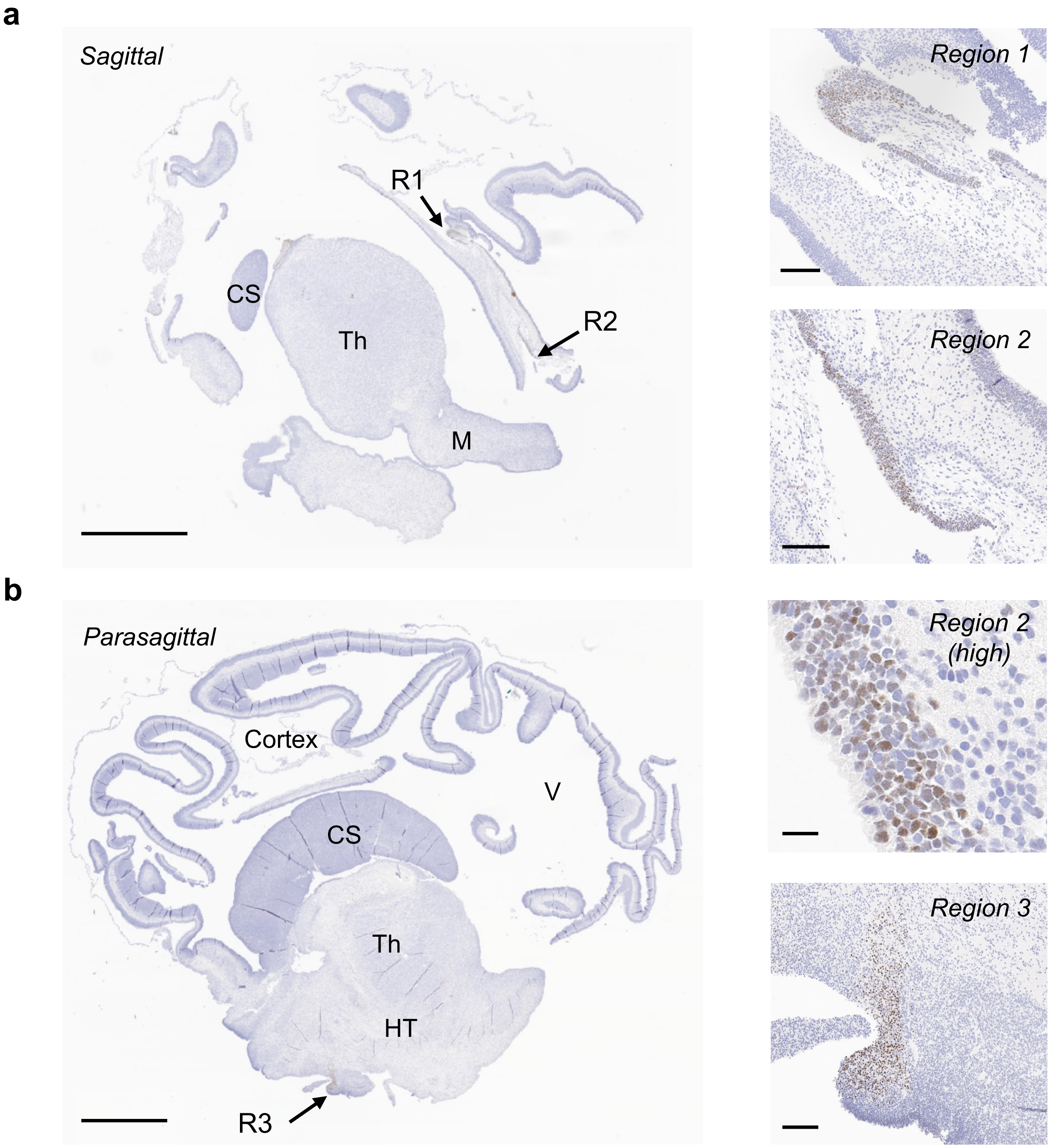

Supplementary Figure 16. Androgen receptor expression by immunohistochemistry in the developing diencephalon and cerebral cortex at 10 weeks post conception. a Sagittal section near the midline. **b** Parasagittal section. Arrows indicate regions of interest shown in higher magnification on the right. Sample 46,XY. Scale bars, 2mm. **c** Higher magnification images of regions of interest. Scale bars, 100 μm; Region 2 high, 20 μm. CS, corpus striatum (ganglionic eminence); HT, hypothalamus; M, midbrain; R, region; Th, thalamus.

Supplementary Figure 17

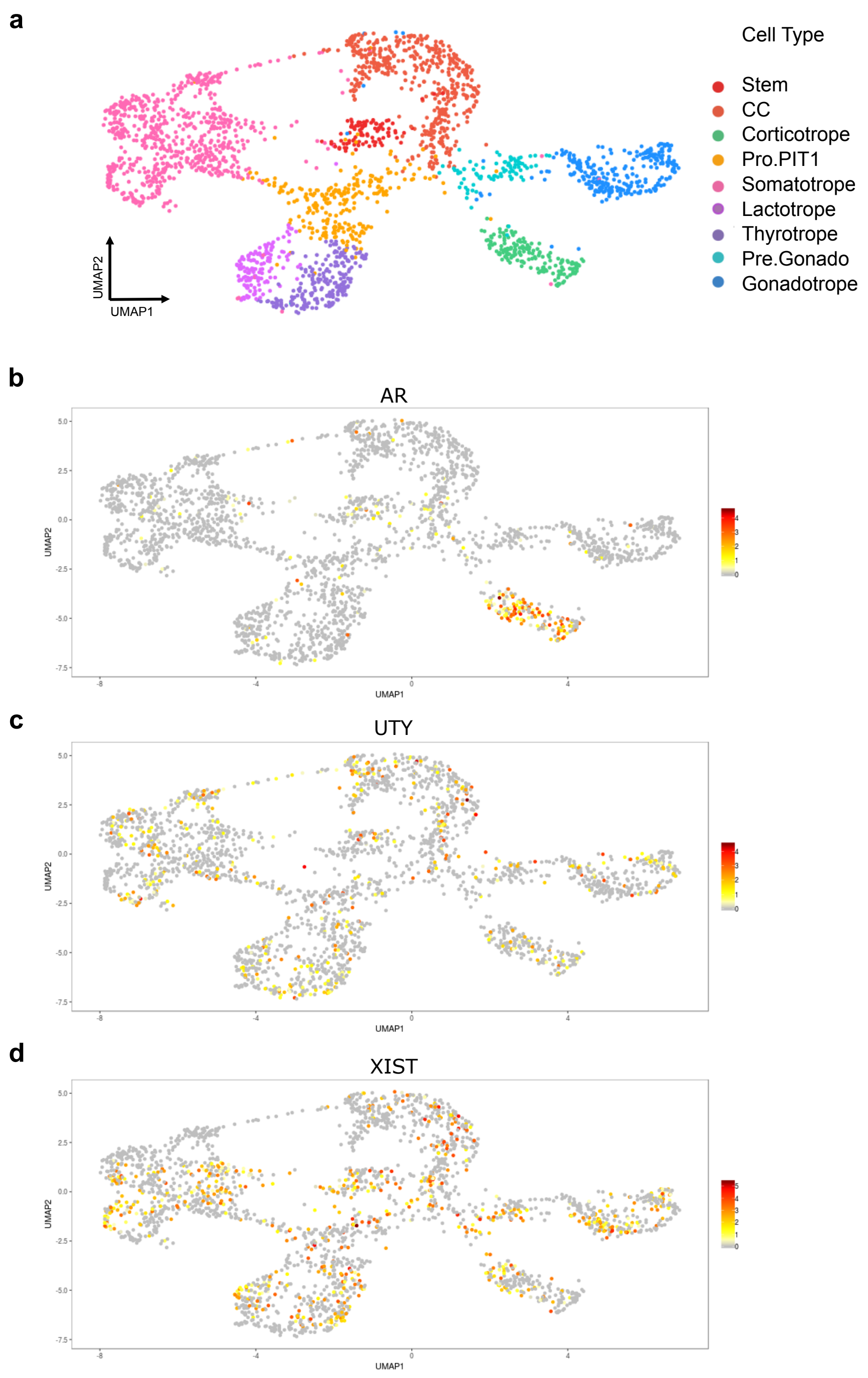

Supplementary Figure 17. Nuclei annotations of single-cell RNA-seq data in the human pituitary. (a) Major cell-type clusters; expression patterns for **(b)** *AR*; **(c)** *UTY*; **(d)** *XIST*. (Zhang S et al., Nat Commun 2020. 11:5275. doi.org/10.1038/s41467-020-19012-4). CC, cell cycle cells.
Pro.PIT1, progenitor cells of PIT1 lineage. Pre.Gonado, precursor cells of gonadotrope. UMAP, (uniform manifold approximation and projection). Data accessed from https://tanglab.shinyapps.io/Human_Fetal_Pituitary_Endocrine_Cells/ (CC BY 4.0, https://creativecommons.org/licenses/by/4.0/)

Supplementary Figure 18

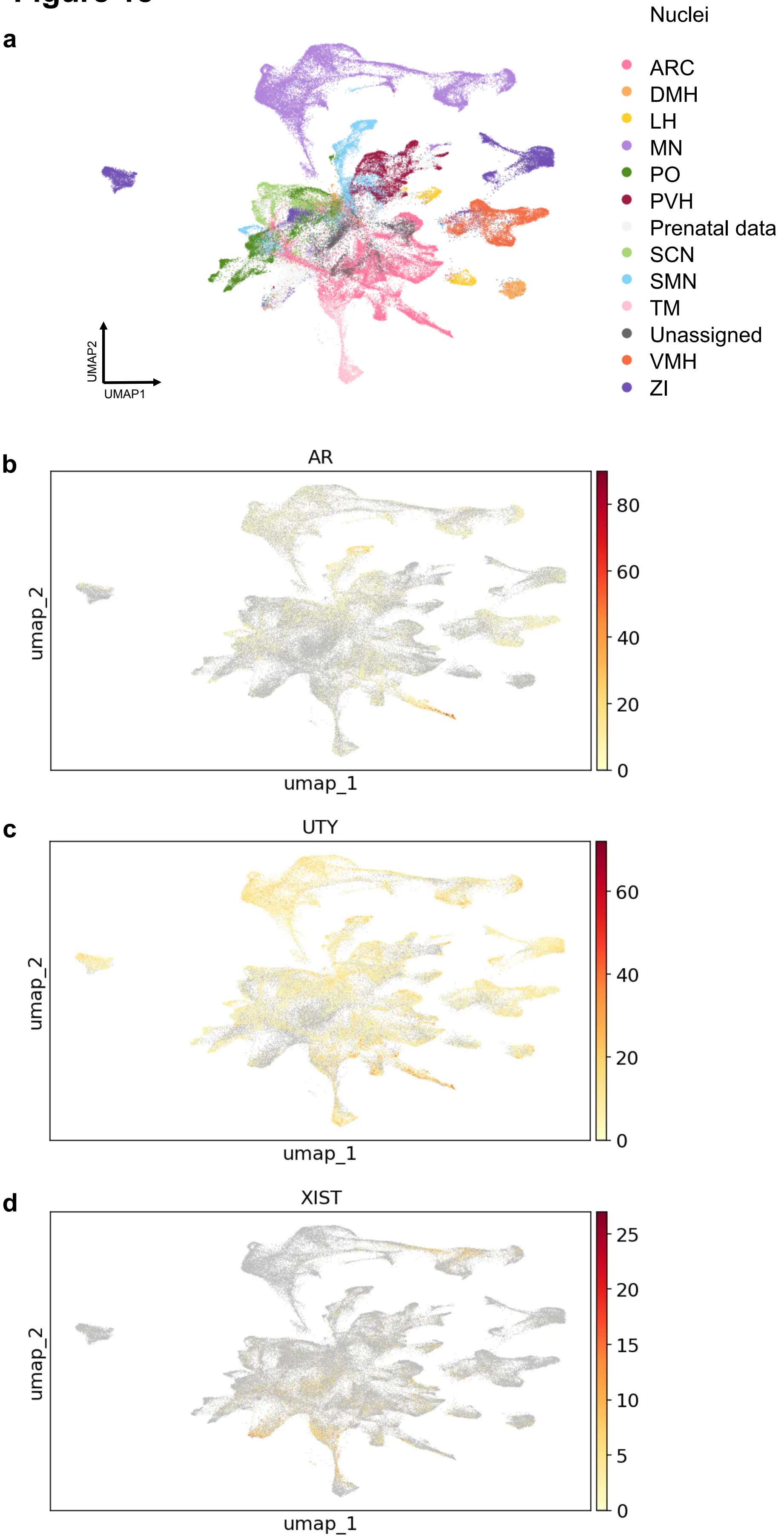

Supplementary Figure 18. Nuclei annotations of single-cell RNA-seq data in the human hypothalamus. (a) Nuclei identified; expression patterns for **(b)** AR; **(c)** UTY; **(d)** XIST. (Herb BR et al., Sci Adv 2023. 9,eadf6251. doi.org/10.1126/sciadv.adf6251). ARC, arcuate nucleus. DMH, dorso-medial nucleus of the hypothalamus. LH, lateral hypothalamus. MN, mammillary nucleus. PO, preoptic area. PVH, paraventricular nucleus of the hypothalamus. SCN, suprachiasmatic nucleus. SMN, supramammillary nucleus. TM, tuberomammillary nucleus. VMH, ventromedial nucleus of the hypothalamus. ZI, zona incerta. UMAP, (uniform manifold approximation and projection). Data accessed from https://nemoanalytics.org/p?l=a856c14e (CC BY-NC 4.0, https://creativecommons.org/licenses/by-nc/4.0/)

Supplementary Figure 18

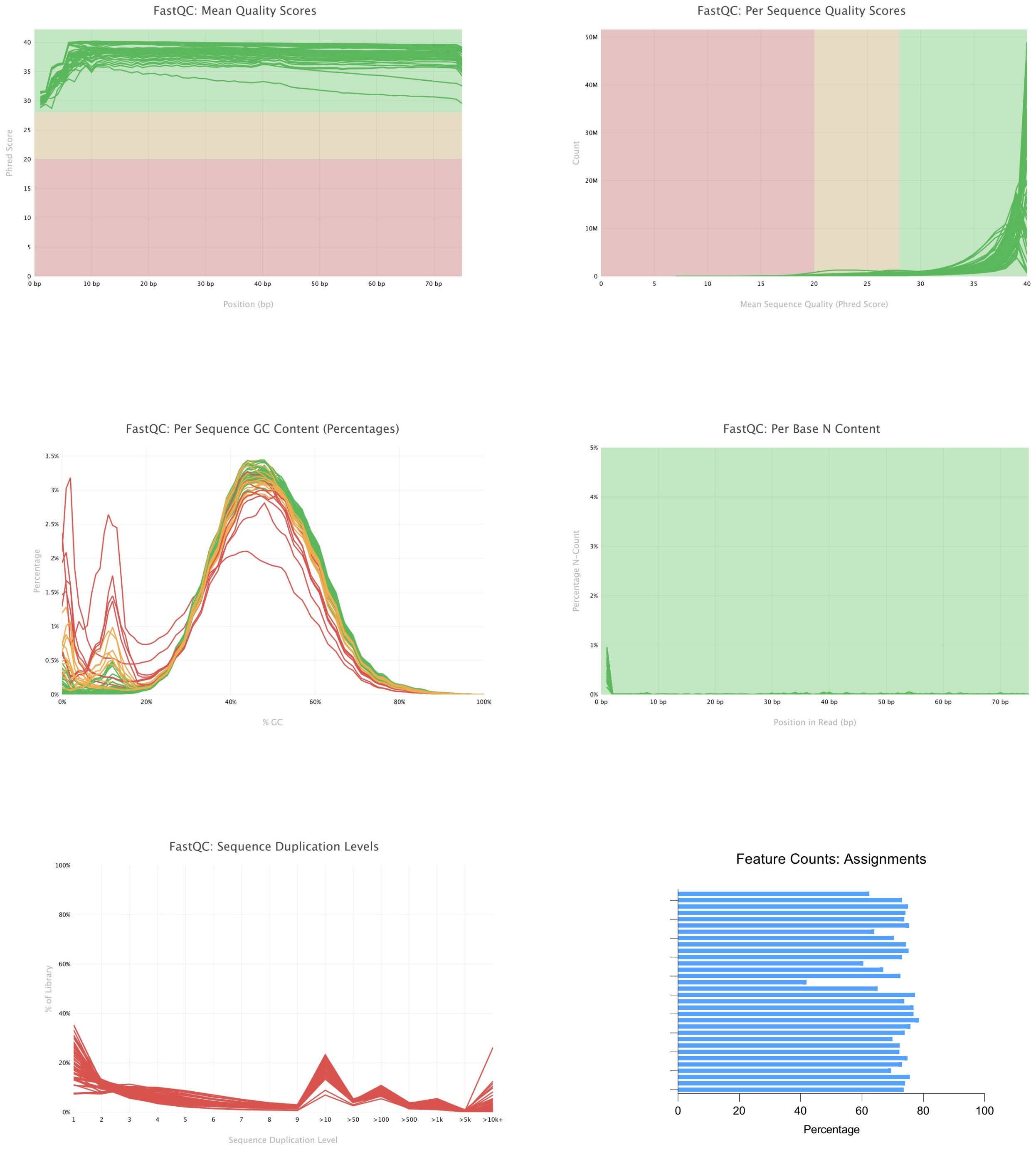

Supplementary Figure 19. MultiQC analysis for the Brain-Seq 1 dataset.

<u>**List of Supplementary Data Files**</u>

Data deposited in Open Science Framework (https://doi.org/10.17605/OSF.IO/AJ3RS)

Supplementary Data 1 for Brain-Seq 1:

Supplementary Data 1.1. Samples used in Brain-Seq 1 dataset.
Supplementary Data 1.2. Top 250 differentially expressed genes, 46,XY versus 46,XX at CS22-23.
Supplementary Data 1.3. Top 250 differentially expressed genes, 46,XX versus 46,XY at CS22-23.
Supplementary Data 1.4. Top 250 differentially expressed genes, 46,XY versus 46,XX at 9wpc.
Supplementary Data 1.5. Top 250 differentially expressed genes, 46,XX versus 46,XY at 9wpc.
Supplementary Data 1.6. Top 250 differentially expressed genes, 46,XY versus 46,XX at 11-12wpc.
Supplementary Data 1.7. Top 250 differentially expressed genes, 46,XX versus 46,XY at 11-12wpc.
Supplementary Data 1.8. Top 250 differentially expressed genes, 46,XY versus 46,XX at 15-17wpc.
Supplementary Data 1.9. Top 250 differentially expressed genes, 46,XX versus 46,XY at 15-17wpc.
Supplementary Data 1.10. Differentially expressed genes, 46,XX 9wpc versus CS22-23.
Supplementary Data 1.11. Differentially expressed genes, 46,XY 9wpc versus CS22-23.
Supplementary Data 1.12. Differentially expressed genes, 46,XX 15-17wpc versus CS22-23.
Supplementary Data 1.13. Differentially expressed genes, 46,XY 15-17wpc versus CS22-23.
Supplementary Data 1.14. Differentially expressed genes, all 46,XY versus all 46,XX.
Supplementary Data 1.15. Normalized counts of all samples in Brain-Seq 1 dataset.

List of Supplementary Data Files

Data deposited in Open Science Framework (https://doi.org/10.17605/OSF.IO/AJ3RS)

Supplementary Data 2 for Brain-Seq 2:

Supplementary data 2.1. Samples used in Brain-Seq 2 dataset.
Supplementary data 2.2. Top 250 differentially expressed genes, 46,XY versus 46,XX at CS22-23.
Supplementary data 2.3. Top 250 differentially expressed genes, 46,XX versus 46,XY at CS22-23.
Supplementary data 2.4. Differentially expressed genes, 46,XY versus 46,XX at 9wpc.
Supplementary data 2.5. Top 250 differentially expressed genes, 46,XX versus 46,XY at 9wpc.
Supplementary data 2.6. Top 250 differentially expressed genes, 46,XY versus 46,XX at 11-12wpc.
Supplementary data 2.7. Top 250 differentially expressed genes, 46,XX versus 46,XY at 11-12wpc.
Supplementary data 2.8. Differentially expressed genes, 46,XY versus 46,XX at 15-17wpc.
Supplementary data 2.9. Differentially expressed genes, 46,XX versus 46,XY at 15-17wpc.
Supplementary data 2.10. Differentially expressed genes, 46,XX 9wpc versus CS22-23.
Supplementary data 2.11. Differentially expressed genes, 46,XY 9wpc versus CS22-23.
Supplementary data 2.12. Differentially expressed genes, 46,XX 15-17wpc versus CS22-23.
Supplementary data 2.13. Differentially expressed genes, 46,XY 15-17wpc versus CS22-23.
Supplementary data 2.14. Differentially expressed genes, all 46,XY versus all 46,XX.
Supplementary data 2.15. Normalized counts of all samples in Brain Seq 2 dataset.

<u>List of Supplementary Data Files</u>

Data deposited in Open Science Framework (https://doi.org/10.17605/OSF.IO/AJ3RS)

Supplementary Data 3 for Control Samples:

Supplementary Data 3.1. Control samples used.
Supplementary Data 3.2. Top 250 differentially expressed genes, skin samples 46,XY versus 46,XX at CS22-23.
Supplementary Data 3.3. Top 250 differentially expressed genes, skin samples 46,XX versus 46,XY at CS22-23.
Supplementary Data 3.4. Top 250 differentially expressed genes, pancreas samples 46,XY versus 46,XX at 9wpc.
Supplementary Data 3.5. Top 250 differentially expressed genes, pancreas samples 46,XX versus 46,XY at 9wpc.
Supplementary Data 3.6. Top 250 differentially expressed genes, liver samples 46,XY versus 46,XX at 11-12wpc.
Supplementary Data 3.7. Top 250 differentially expressed genes, liver samples 46,XX versus 46,XY at 11-12wpc.
Supplementary Data 3.8. Top 250 differentially expressed genes, kidney samples 46,XY versus 46,XX at 15-17wpc.
Supplementary Data 3.9. Top 250 differentially expressed genes, kidney samples 46,XX versus 46,XY at 15-17wpc.

<u>List of Supplementary Data Files</u>

Data deposited in Open Science Framework (https://doi.org/10.17605/OSF.IO/AJ3RS)

Supplementary Data 4 for Figures:

Supplementary Data 4.1. Figure 5c. Log2 fold change values of *PCDH11Y* in both brain datasets compared to controls across each developmental stage.
Supplementary Data 4.2. Figure 5d. qRT-PCR data points and analysis of *PCDH11Y* in 46,XY brain cortex across key developmental stages, and compared to control.
Supplementary Data 4.3. Figure 5e. Expression of *PCDH11Y* in the Human Protein Atlas Consensus dataset for adult brain, (data accessed and downloaded from https://www.proteinatlas.org). nTPM, normalized transcript per million.
Supplementary Data 4.4. Supplementary Figure 8. Number of differentially expressed genes (DEGs) (46,XX versus 46,XY) in the Brain-Seq 1 and Brain-Seq 2 datasets with developmental stage.
