# Supplementary material for: Transcriptomic sex differences in early human fetal brain development
Source: Commun Biol. 2025 Apr 25;8:664. doi: 10.1038/s42003-025-08070-3 (PMC12032161; doi:10.1038/s42003-025-08070-3)
Supplement: Supplementary file 2 — Description of Additional Supplementary Files [file 42003_2025_8070_MOESM2_ESM.docx]

Description of Additional Supplementary Files

**File name:** Supplementary Data 1

**Description:**

- Supplementary Data 1.1: Samples used in Brain-Seq 1 dataset.
- Supplementary Data 1.2: Top 250 differentially expressed genes, 46,XY versus 46,XX at CS22-23.
- Supplementary Data 1.3: Top 250 differentially expressed genes, 46,XX versus 46,XY at CS22-23.
- Supplementary Data 1.4: Top 250 differentially expressed genes, 46,XY versus 46,XX at 9wpc.
- Supplementary Data 1.5: Top 250 differentially expressed genes, 46,XX versus 46,XY at 9wpc.
- Supplementary Data 1.6: Top 250 differentially expressed genes, 46,XY versus 46,XX at 11-12wpc.
- Supplementary Data 1.7: Top 250 differentially expressed genes, 46,XX versus 46,XY at 11-12wpc.
- Supplementary Data 1.8: Top 250 differentially expressed genes, 46,XY versus 46,XX at 15-17wpc.
- Supplementary Data 1.9: Top 250 differentially expressed genes, 46,XX versus 46,XY at 15-17wpc.
- Supplementary Data 1.10: Differentially expressed genes, 46,XX 9wpc versus CS22-23.
- Supplementary Data 1.11: Differentially expressed genes, 46,XY 9wpc versus CS22-23.
- Supplementary Data 1.12: Differentially expressed genes, 46,XX 15-17wpc versus CS22-23.
- Supplementary Data 1.13: Differentially expressed genes, 46,XY 15-17wpc versus CS22-23.
- Supplementary Data 1.14: Differentially expressed genes, all 46,XY versus all 46,XX.
- Supplementary Data 1.15: Normalized counts of all samples in Brain-Seq 1 dataset.

**File name:** Supplementary Data 2

**Description:**

- Supplementary data 2.1: Samples used in Brain-Seq 2 dataset.
- Supplementary data 2.2: Top 250 differentially expressed genes, 46,XY versus 46,XX at CS22-23.
- Supplementary data 2.3: Top 250 differentially expressed genes, 46,XX versus 46,XY at CS22-23.
- Supplementary data 2.4: Differentially expressed genes, 46,XY versus 46,XX at 9wpc.
- Supplementary data 2.5: Top 250 differentially expressed genes, 46,XX versus 46,XY at 9wpc.
- Supplementary data 2.6: Top 250 differentially expressed genes, 46,XY versus 46,XX at 11-12wpc.
- Supplementary data 2.7: Top 250 differentially expressed genes, 46,XX versus 46,XY at 11-12wpc.
- Supplementary data 2.8: Differentially expressed genes, 46,XY versus 46,XX at 15-17wpc.
- Supplementary data 2.9: Differentially expressed genes, 46,XX versus 46,XY at 15-17wpc.
- Supplementary data 2.10: Differentially expressed genes, 46,XX 9wpc versus CS22-23.
- Supplementary data 2.11: Differentially expressed genes, 46,XY 9wpc versus CS22-23.
- Supplementary data 2.12: Differentially expressed genes, 46,XX 15-17wpc versus CS22-23.
- Supplementary data 2.13: Differentially expressed genes, 46,XY 15-17wpc versus CS22-23.
- Supplementary data 2.14: Differentially expressed genes, all 46,XY versus all 46,XX.
- Supplementary data 2.15: Normalized counts of all samples in Brain Seq 2 dataset.

**File name:** Supplementary Data 3

**Description:**

- Supplementary Data 3.1: Control samples used.
- Supplementary Data 3.2: Top 250 differentially expressed genes, skin samples 46,XY versus 46,XX at CS22-23.
- Supplementary Data 3.3: Top 250 differentially expressed genes, skin samples 46,XX versus 46,XY at CS22-23.
- Supplementary Data 3.4: Top 250 differentially expressed genes, pancreas samples 46,XY versus 46,XX at 9wpc.
- Supplementary Data 3.5: Top 250 differentially expressed genes, pancreas samples 46,XX versus 46,XY at 9wpc.
- Supplementary Data 3.6: Top 250 differentially expressed genes, liver samples 46,XY versus 46,XX at 11-12wpc.
- Supplementary Data 3.7: Top 250 differentially expressed genes, liver samples 46,XX versus 46,XY at 11-12wpc.
- Supplementary Data 3.8: Top 250 differentially expressed genes, kidney samples 46,XY versus 46,XX at 15-17wpc.
- Supplementary Data 3.9: Top 250 differentially expressed genes, kidney samples 46,XX versus 46,XY at 15-17wpc.

**File name:** Supplementary Data 4

**Description:**

- Supplementary Data 4.1: Figure 5c. Log2 fold change values of PCDH11Y in both brain datasets compared to controls across each developmental stage.
- Supplementary Data 4.2: Figure 5d. qRT-PCR data points and analysis of PCDH11Y in 46,XY brain cortex across key developmental stages, and compared to control.
- Supplementary Data 4.3: Figure 5e. Expression of PCDH11Y in the Human Protein Atlas Consensus dataset for adult brain, (data accessed and downloaded from https://www.proteinatlas.org). nTPM, normalized transcript per million.
- Supplementary Data 4.4: Supplementary Figure 8. Number of differentially expressed genes (DEGs) (46,XX versus 46,XY) in the Brain-Seq 1 and Brain-Seq 2 datasets with developmental stage.
