# Supplementary material for: Transcriptomic sex differences in early human fetal brain development
Source: Commun Biol. 2025 Apr 25;8:664. doi: 10.1038/s42003-025-08070-3 (PMC12032161; doi:10.1038/s42003-025-08070-3)
Supplement: Supplementary file 7 — Reporting summary [file 42003_2025_8070_MOESM7_ESM.pdf]

nature portfolio

Corresponding author(s): Federica Buonocore
Last updated by author(s): 04/04/2025

# Reporting Summary

Nature Portfolio wishes to improve the reproducibility of the work that we publish. This form provides structure for consistency and transparency in reporting. For further information on Nature Portfolio policies, see our Editorial Policies and the Editorial Policy Checklist.

## Statistics

For all statistical analyses, confirm that the following items are present in the figure legend, table legend, main text, or Methods section.

| n/a | Confirmed | |
|---|---|---|
| ☐ | ☒ | The exact sample size (*n*) for each experimental group/condition, given as a discrete number and unit of measurement |
| ☐ | ☒ | A statement on whether measurements were taken from distinct samples or whether the same sample was measured repeatedly |
| ☐ | ☒ | The statistical test(s) used AND whether they are one- or two-sided<br>*Only common tests should be described solely by name; describe more complex techniques in the Methods section.* |
| ☒ | ☐ | A description of all covariates tested |
| ☐ | ☒ | A description of any assumptions or corrections, such as tests of normality and adjustment for multiple comparisons |
| ☐ | ☒ | A full description of the statistical parameters including central tendency (e.g. means) or other basic estimates (e.g. regression coefficient) AND variation (e.g. standard deviation) or associated estimates of uncertainty (e.g. confidence intervals) |
| ☐ | ☒ | For null hypothesis testing, the test statistic (e.g. *F*, *t*, *r*) with confidence intervals, effect sizes, degrees of freedom and *P* value noted<br>*Give P values as exact values whenever suitable.* |
| ☒ | ☐ | For Bayesian analysis, information on the choice of priors and Markov chain Monte Carlo settings |
| ☒ | ☐ | For hierarchical and complex designs, identification of the appropriate level for tests and full reporting of outcomes |
| ☒ | ☐ | Estimates of effect sizes (e.g. Cohen's *d*, Pearson's *r*), indicating how they were calculated |

*Our web collection on statistics for biologists contains articles on many of the points above.*

## Software and code

Policy information about availability of computer code

| | |
|---|---|
| Data collection | No software was used |
| Data analysis | Standard open source packages were used for analysis in R as outlined in the Methods. GraphPad (v8.4.3) was used for qRT-PCR analysis. |

For manuscripts utilizing custom algorithms or software that are central to the research but not yet described in published literature, software must be made available to editors and reviewers. We strongly encourage code deposition in a community repository (e.g. GitHub). See the Nature Portfolio guidelines for submitting code & software for further information.

## Data

Policy information about availability of data

All manuscripts must include a data availability statement. This statement should provide the following information, where applicable:

- Accession codes, unique identifiers, or web links for publicly available datasets
- A description of any restrictions on data availability
- For clinical datasets or third party data, please ensure that the statement adheres to our policy

The datasets generated and analyzed during the current study can be found in the following links:
1. Brain-Seq1 (bulk RNA-seq): ArrayExpress/Biostudies (accession number E-MTAB-13662; https://www.ebi.ac.uk/biostudies/arrayexpress/studies)
2. Brain-Seq2 (bulk RNA-seq): https://www.ebi.ac.uk/gxa/experiments/E-MTAB-4840/Downloads (ref 26)
3. Control samples (bulk RNA-seq): ArrayExpress/Biostudies (accession number E-MTAB-13673; https://www.ebi.ac.uk/biostudies/arrayexpress/studies) (Ref 52)

4. Supplementary Data files (Brain-Seq1, Brain-Seq2; Control samples; Figure 5 and Supplementary Figure 8) have been deposited in Open Science Framework (https://doi.org/10.17605/OSF.IO/AJ3RS) (Ref 57)

## Research involving human participants, their data, or biological material

Policy information about studies with human participants or human data. See also policy information about sex, gender (identity/presentation), and sexual orientation and race, ethnicity and racism.

| | |
|---|---|
| Reporting on sex and gender | The whole purpose of this work is to compare tissues with different karyotypes (46,XX; 46,XY). As this is fetal material, we use karyotype as a proxy for biological sex, and avoid terms such as "male" or "female". We do not use "gender" in a non-biological context. All groups were balanced in number and analysis, as this was the main focus of the study. The comparisons are between different karyotypes. |
| Reporting on race, ethnicity, or other socially relevant groupings | We cannot report on race, ethnicity or social-economic variables as all tissues that are provided by the Human Developmental Biology Resource are pseudononymised, and these data are not available. |
| Population characteristics | The samples are all fetal tissue samples with very clearly defined developmental age and karyotype/sex. |
| Recruitment | The 46,XY and 46,XX samples were obtained randomly to match the developmental stages under consideration. There was no knowledge of specific characteristics, or collection variables that that might influence or bias the outcome. In addition, QC of certain aspects was undertaken (eg RNA integrity) post hoc, and no relevant differences were seen. These findings are now all included in the manuscript. |
| Ethics oversight | UK Research Ethics Committee references: 08/H0712/34+5, 18/LO/0822, 08/H0906/21+5, 18/NE/0290; HDBR project references 200332, 200655 |

Note that full information on the approval of the study protocol must also be provided in the manuscript.

# Field-specific reporting

Please select the one below that is the best fit for your research. If you are not sure, read the appropriate sections before making your selection.

☒ Life sciences ☐ Behavioural & social sciences ☐ Ecological, evolutionary & environmental sciences

For a reference copy of the document with all sections, see nature.com/documents/nr-reporting-summary-flat.pdf

# Life sciences study design

All studies must disclose on these points even when the disclosure is negative.

| | |
|---|---|
| Sample size | For bulk RNA seq studies, a minimum of 3 samples is usually considered standard for assessing differential gene expression. Here we were able to include groups of 4 samples in each comparison (karyotype, stage/age), as transcriptomic sex differences can be more subtle and adjustment for multiple comparisons are needed. We also removed any filter for log2 fold change in the revised version of this manuscript, using just adjusted p value, as we wanted to avoid false negatives and it was felt we were being too stringent in the Reviewers' opinion. Furthermore, n the revised manuscript, we have generated a "global" whole group of n=16 for each karyotypes (46,XX; 46,XY) in each dataset to increase power, and used several different approaches to data analysis and presentation, as suggested by Reviewers. These approaches were useful although the final results changed little. All data are available in Supplementary files and in repositories. |
| Data exclusions | No data were excluded. |
| Replication | 1) Bulk RNA seq data were verified by including TWO COMPLETE MATCHED REPLICATION STUDIES (n=32 samples each), which showed consistent patterns (e.g. androgen receptor gene expression over time) but which also prevented us reporting likely false positive findings or "noise". We feel that this was a very stringent and powerful approach.<br>2) qRT-PCR of key genes identified to replicate differences seen in bulk analysis (eg PCDH11Y, PCDH11X).<br>3) Immunohistochemistry to analyze expression of AR at the protein level. and in localised regions of the fetal brain. |
| Randomization | All 46,XX and 46,XY samples that were available were included and age matched on a random basis without any available insight into other characteristics. |
| Blinding | Blinding was not relevant to this study and samples were included randomly based on availability. In the manuscript revised form the origibal NATCOMMS submission, much more detailed methods are provided about sampling and operating pipelines. |

# Reporting for specific materials, systems and methods

We require information from authors about some types of materials, experimental systems and methods used in many studies. Here, indicate whether each material, system or method listed is relevant to your study. If you are not sure if a list item applies to your research, read the appropriate section before selecting a response.

## Materials & experimental systems

| n/a | Involved in the study | |
|---|---|---|
| ☐ | ☒ | Antibodies |
| ☒ | ☐ | Eukaryotic cell lines |
| ☒ | ☐ | Palaeontology and archaeology |
| ☒ | ☐ | Animals and other organisms |
| ☒ | ☐ | Clinical data |
| ☒ | ☐ | Dual use research of concern |
| ☒ | ☐ | Plants |

## Methods

| n/a | Involved in the study | |
|---|---|---|
| ☒ | ☐ | ChIP-seq |
| ☒ | ☐ | Flow cytometry |
| ☒ | ☐ | MRI-based neuroimaging |

# Antibodies

Antibodies used

For immunohistochemistry, primary androgen receptor (AR, NR3C4) rabbit monoclonal antibody (Abcam ab108341 ChIP grade, 1:50 dilution, HIER2 for 20 mins)

Validation

1) The manufacturers data sheet (https://doc.abcam.com/datasheets/active/ab108341/en-us/androgen-receptor-antibody-er1792-chip-grade-ab108341.pdf) shows specificity for the antibody on testis, prostate cancer etc
2) The antibody has be used in 79 published papers in this resource
3) Our department of research histopathology optimized the antibody on independent control samples (based on human tissue atlas expression) and the results and protocols were independently and validated by an experienced histopathologist. Control data for testis epididymis are shown in Figure 7.

# Plants

Seed stocks

n/a

Novel plant genotypes

n/a

Authentication

n/a
